# Acceptance of hearing protection aids in members of an instrumental and voice music band

**DOI:** 10.1016/S1808-8694(15)31175-7

**Published:** 2015-10-19

**Authors:** Maria Helena Mendes, Thais Catalani Morata, Jair Mendes Marques

**Affiliations:** 1 M.Sc. in Communications Disorders, Speech and Hearing Therapy; 2 PhD in Communications Disorders - University of Cincinnati, Professor - Universidade Tuiuti do Paraná; 3 PhD in Geodesic Sciences - Universidade Federal do Paraná, Professor - Universidade Tuiuti do Paraná. Universidade Tuiuti do Paraná

**Keywords:** musicians, music induced hearing loss, hearing protection

## Abstract

**Summary:**

There are barriers to effective hearing protection among musicians.

**Aim:**

To investigate the acceptance of hearing protection aids in members of an instrumental and voice music band.

**Material and method:**

A prospective study of 34 members of the Municipal Indaial Band. Sound pressure levels were measured during a rehearsal, indicating mean levels ranging from 96.4 dB(A) to 106.9 dB(A). Subjects answered questionnaires and underwent audiometry. They attended a lecture in which folders and hearing protection aids were provided; subjects were asked to try using the protectors for 3 months.

**Results:**

At the end of the study period, 56.2% reported not liking hearing protection, while 43.7 % accepted such protection. The most common complaints were discomfort with sounds (58.8 %) and tinnitus (47%). 77.1% said that music might cause hearing impairment. A statistically significant difference was observed in the right ear at 4 and 6 kHz and at the left ear in 3, 4 and 6 kHz when median thresholds were compared with those from unexposed controls.

**Conclusion:**

Although most subjects seemed aware of the risk, few took preventive measures against hearing loss. This suggests the need for periodic educational campaigns and specific legislation tailored to music professionals.

## INTRODUCTION

We usually do not think of music as being a noise, but rather as a pleasant sound. However, when played loud, it may become a potential threat to the human ear^1^.

There are some differences between music and noise. In music, the temporal pattern is floating, the main frequencies are low, damped even further by the stapes, and it is usually pleasant. Noise, however, has a continuous temporal pattern, the main frequencies are high and it is unpleasant to the human ear.

The association between noise exposure and occupational hearing loss has been described for more than one century, however, it was only after the 60's that some researchers showed some concern with the effects of music to hearing^2^,^3^.

Studies have shown hearing loss in rock band members, sound cars, orchestras, ballroom bands, or even in individual music instruments training^4^, ^5^, ^6^, ^7^, ^8^.

Hearing loss preventive measures have been suggested to musicians based on numerous scientific researches, such as: acoustic treatment of rehearsing environments, audiologic follow up and individual hearing protection, among others^6^, ^7^, ^8^.

Today, musicians from Brazil and abroad have been offered specific in-the-ear protection devices. They allow for damping balance in all the frequencies, with an uniform sound reduction, avoiding the effect of occlusion and, consequently, sound distortion. Options range from simple models of standard size that offer different music damping, to customized ear protection devices.

The current investigation aims at studying the acceptance of individual hearing protection devices by band members and singers during their research and performances.

## MATERIALS AND METHODS

This investigation was carried out in the city of Indaial, involving the members of the City Band. This band is made up of 36 members, 6 women and 30 men, with ages varying between 19 and 76 years, mean age of 40 years. This study was a historical cohort with cross-sections.

The band has the following members: vocal (8) persons, keyboards (1), saxophone (5), drums and percussion (3), clarinet (2), tube (1), electrical guitar (1), bass (1), trombone (5), trumpet (6), transversal flute (1), conductor (1), sound mixer (1). The band is paid by the Town Hall of Indaial, and they have an engagement with the Town Hall of 4 times per month between rehearsals and performances. We had 34 individuals participating in this investigation after having been duly informed about it and signing the informed consent form. The present study was approved by the Ethics Committee of the Tuiuti University of Paraná, under protocol # 001/2005.

The participants were submitted to a questionnaire with open questions and multiple-choice questions with: identification, type of musical instrument the person plays, for how long the person had been playing the instrument (as a musician, singer, or sound mixer operator), how often they rehearse and perform, individual practice, previous or concomitant exposure to industrial noise, auditory complaints, family history of hearing loss, diseases, medication use and hearing care.

The questionnaire was applied by the researcher prior to the auditory assessment.

Audiologic assessment of the band members was carried out after an acoustic rest of fourteen hours, under the following routine:

a) Visual inspection of the external acoustic meatus: for that we used a Welch Allyn otoscope aiming at checking for some obstruction that would prevent the test from being carried out. If there was any alteration the subjects were referred to otolaryngological evaluation and then they returned for their audiologic evaluation.
b) Audiometry: Tonal threshold audiometry was carried out in a sound proof booth, with a clinical audiometer - MAICO, model MA-41, TDH39 headphone, checked according to ISO 8253-1 standard and Resolution 296/03 of the Federal Board of Speech and Hearing Therapy. The goal of tonal audiometry was to determine the air conduction hearing thresholds in the frequencies of 250, 500, 1000, 2000, 3000, 4000, 6000 e 8000 Hz, and bone conduction in the frequencies of 500, 1000, 2000, 3000 e 4000 Hz.

In order o classify the hearing thresholds of band members we used the criteria of audiometric alterations proposed by Fiorini (1994)^9^:

– Audiograms suggesting normal hearing: individuals who had all bilateral thresholds within the range of 25dBHL.
– Audiograms suggesting noise induced hearing loss (NIHL): individuals who had hearing loss configuration (thresholds above 25dBHL) in the frequencies of 6 and/or 4 and/or 3 kHz).
– Audiograms with other types of classification: individuals who had hearing loss thresholds above 25dBHL, and whose audiometric configuration did not match previous classifications.

In order to analyze the audiograms we excluded 11 individuals (32.3%) exposed to other noisy professional activities that are not related to music (2 trumpet, 1 trombone, 1 tube, 1 clarinet, 2 saxophone), as well as individuals with conductive hearing loss (1 trumpet, 1 clarinet), mixed hearing loss (1 drums and percussion), and one individual with profound unilateral sensorineural hearing loss (1 drums and percussion).

Of the population analyzed in this study we had 23 individuals in the study group (67.6%), exposed to music alone, who had their hearing thresholds compared to those in the control group, paired according to gender and age.

For the control group we selected^23^ individuals without exposure to noise, collected in the Laboratory of Audiology at the University of Tuiuti - Paraná State^10^.

We calculated the median value of the auditory threshold in the right and left ears of the experimental and the control groups. In order to compare auditory thresholds from the control and experimental groups in both the right and the left ears, we used the Wilcoxon statistical test, and 5% (= 0.05) was the level of significance. There was no statistically significant difference between the two groups as to the age of the individuals.

**Figure 1:**
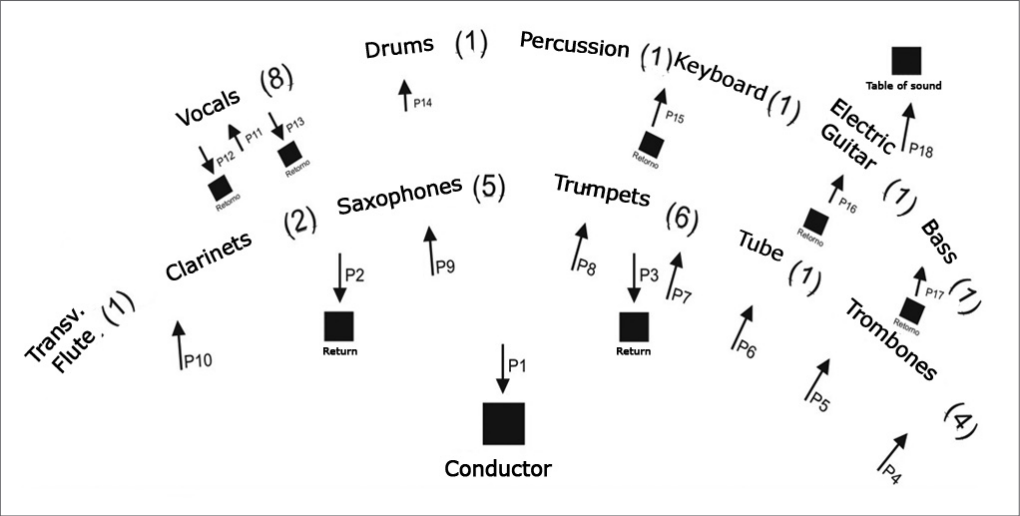
Band configuration in the rehearsal room with Sound Pressure Levels measurement points

Sound pressure level was assessed by an engineer during a band rehearsal. The measurements were taken by means of a sound pressure level measuring device - QUEST-2700, using the fast response mode (F), and the dB scale (A), filtering method, which come close to the reception characteristics of the human ear. The rehearsal room with reverberating characteristics was divided in 18 points of measurement located in: P1(conductor), P2 (feedback+clarinets), P3 (feedback+trumpets), P4 (trombones), P5 (trombones), P6 (trombones+trumpets), P7 (trumpets), P8 (trumpets), P9 (saxophones), P10 (clarinets+flute), P11 (vocals), P12 (vocals+feedback), P13 (vocals+feedback), P14 (drums), P15 (percussion+keyboar ds+feedback), P16 (guitar+feedback), P17 (bass+feedback), P18 (sound mixer). (Figure 1)

We stress that the assessment of the sound pressure levels to which the musicians are exposed to is complex and variable because it depends on environmental conditions in which the orchestra performance happens. Therefore, the measurements carried out have a merely illustrative characteristic of the potential risk associated with the exposure for these musicians. We instructed the musicians about the measurements that we were going to carry out, risks of hearing impairment with periodic exposure to intense music, basic hearing anatomy and physiology, as well as instructions and training to help them use personal protection equipment (PPE) during rehearsals and performances. During this talk we handed out to the band members the PPE, brochures with all the information necessary to properly use the PPE, information about its importance and hearing anatomy and physiology. The musicians were required to use PPE for a period of three months. The author prepared all the content of the talk and the brochures based on educational material created by the NHCA (National Hearing Conservation Association)^11^, ^12^, ^13^ and by others^14^, ^15^, ^16^.

**Figure 2:**
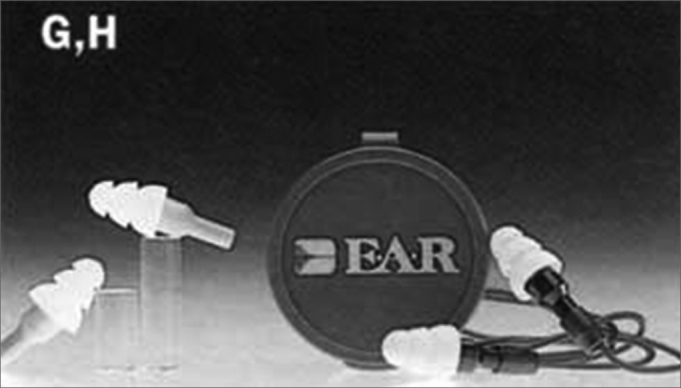
Single size ear protection device - ER 20 E.A.R. Ultratech Earplugs

Source: http://www.drumchops.com

The hearing protection device selected was of the model ER-20 from E.A.R. Ultratech Earplugs (Figure 2). Table 1 shows its damping by frequency range. These protection equipment were distributed to the musicians, singers and sound mixer operator without any cost for them. Choice criteria for this PPE was based on the following aspects: constant damping, single size, it does not require technical personnel for its making, acquisition cost was lower when compared to customized hearing protection devices, and the damping proposal offered by the manufacturer is adequate to the needs of the group studied.

**Table 1:** Damping curve for the auditory protection device E.A.R Ultratech ER-20

Damping in dB	Frequency (Hz)							
	63	125	250	500	1000	2000	4000	8000
Mean Damping	14,3	15,3	18,1	20,8	21,8	26,3	21,5	27,0
Standard Deviation	3,3	2,9	3,6	4,3	3,5	3,0	3,2	4,7
Protection Used	11,0	12,3	14,5	16,4	18,3	23,3	18,3	22,3

SOURCE: www.weststarmusic.com/html/hearing_protection.html

After the three months proposed for PPE use in rehearsals and performances, the participants answered another questionnaire intended to check for hearing protection device use and acceptance.

## RESULTS

As we measured sound pressure level in the rehearsal room, we observed mean values of 96.4 and 106.9 dB (A), and the highest levels of sound pressure were associated with the trumpet. Results from the measurements attained from the measurement points listed above, and also the mean values obtained are all on Table 2.

**Table 2:** Sound Pressure Levels measuring points during band rehearsal

Measuring Points	SPL/dB* (Peak)	SPL/dB* (Mean value)
P1 (Conductor)	107.9	103.6
P2 (Clarinets' Return sound)	107.9	103.6
P3 (Trumpets' Return sound	107.9	103.6
P4 (Trombones)	105.8	101.6
P5 (Trombones)	106.8	103.3
P6 (Trombones/Trumpets)	110.6	104.3
P7 (Trumpets)	110.9	106.9
P8 (Trumpets)	108.8	104.3
P9 (Saxophones)	107.1	101.5
P10 (Clarinets/Flute)	105.5	102.1
P11 (Vocals)	104.3	100.1
P12 (Vocals Return sound)	101.1	96.4
P13 (Vocals Return sound)	104.3	98.1
P14 (Drums)	104.1	102.2
P15 (Percussion/Keyboard/Return sound)	103.1	98.7
P16 (Electric Guitar/Return sound)	104.8	101.4
P17 (Bass/ Return sound)	104.6	101.3
P18 (Mixer)	98.7	98.7

* ound Pressure Level/decibel

Of the 34 individuals in this research paper, 28 (80%) were males and 6 (17.1%) were females, with ages varying between 19 and 76 years.

**Graph 1:**
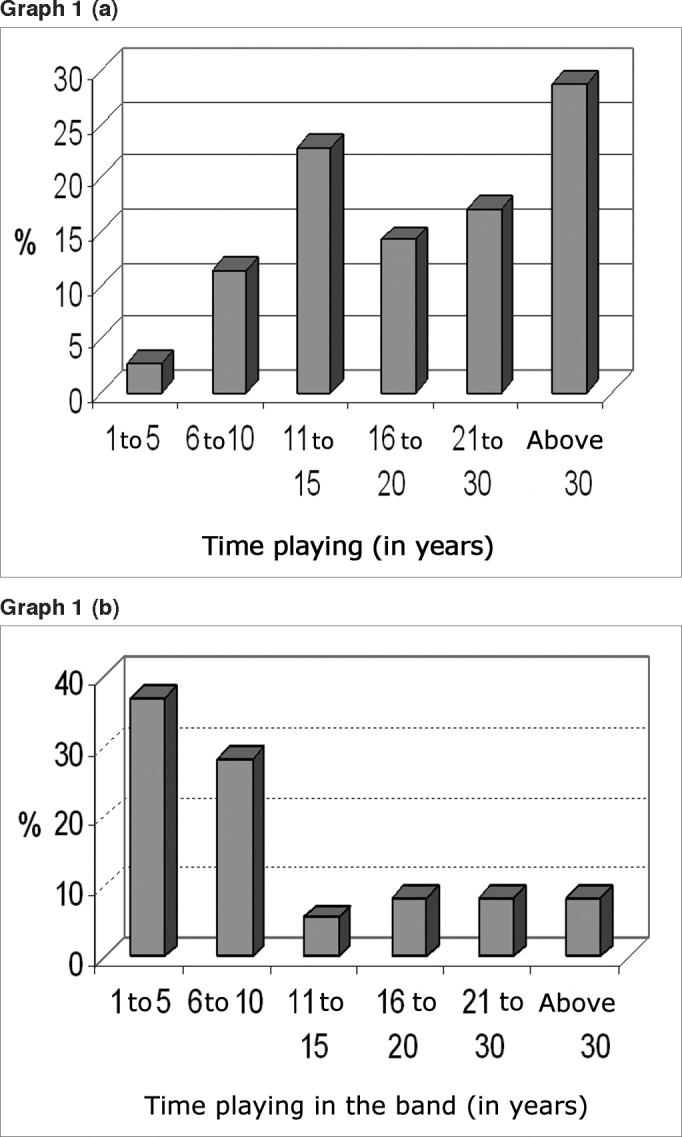
Distribution of individuals according to (a) time playing and (b) time playing in the Municipal Band of Indaial

In Graph 1 (a), we can find the time these people spend practicing and playing music, while in Graph 1 (b) we find the time they have spent playing in this specific band, the Municipal Band of Indaial.

In Chart 1, we see the exposure of these individuals in the study to other music-related activities (23 people, 67.6%), like in other scenarios involving exposure to high sound pressure levels (11 subjects, 32.3%).

**Chart 1:** Exposure to other musical settings and other noisy activities

Other Musical Settings	Other Noisy Activities
Musician of ballroom bands	Forklift operation
Music professor	Wiring
Musician of other instrumental bands	Machine operation
Musicians of symphonic orchestra	Shooting practice
Conductor	Weaving
Radio host and professional speaker	Metallurgy
Choir singing	Heavy mechanics
Singer of ballroom bands	Kart-car races organizer
Individual rehearsals/type	Waiter in dance clubs

The hearing complaints of these musicians, singers and sound mixer operators were sound discomfort (58.8%), tinnitus (47%), hearing loss (25.7%) and a feeling of blocked ear (4%).

When questioned about the possibility of music causing hearing damage, 27 individuals (77.1%) agreed with it and 7 (20%) answered negatively to it. Moreover, 9 subjects (25.7%) complained of hearing loss. Only 9 individuals (25.7%) stated they took some care of their hearing and 25 (71.4%) were not careful with their hearing. Among the care most often mentioned are: the use of PPE at work in factories, as in the practice of shooting with guns, use of cotton ball during exposure to loud music, play softly, avoid loud noises.

**Graph 2:**
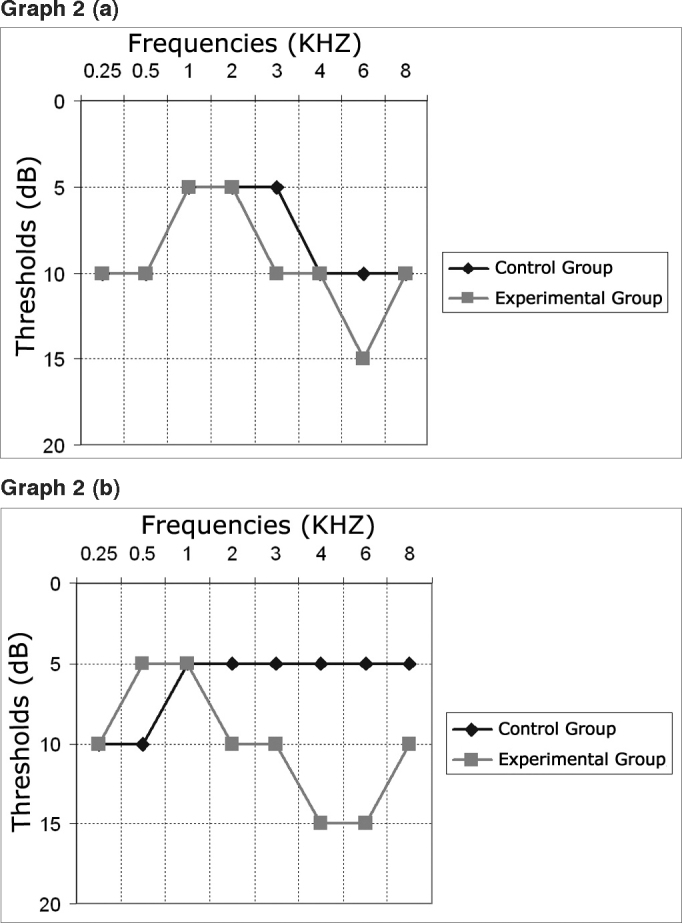
Medians of the Control and Experimental Groups on the Right (a) and Left (b) Ears

We calculated the median values of auditory thresholds in the right and left ears of the experimental and control groups, detailed on Graph 2 (a) and (b). We performed the Wilcoxon test considering 5% (= 0.05) as a level of significance between the thresholds of both groups if p <. We notice that the difference is significant in the frequencies of 4 and 6 kHz in the right ear, and in

the frequencies of 3, 4 and 6 kHz in the left ear, because in these cases p < = 0.05.

According to the criterion used for audiometric alteration13 we found results suggesting hearing loss among the singers (2), trumpet (3), trombone (1), saxophone (2) and guitar (1).

In order to analyze the acceptance related to wearing a hearing protection device or PPE, 32 individuals participated, of the 34 members of the band who participated in the study, 2 individuals were not present to answer the survey questionnaire, and were, therefore, taken off the study.

When asked whether or not they liked wearing the hearing protection device, 18 of them (56.2%) answered no, while 14 (43.7%) said yes. The reasons for them liking or not the hearing protection are expressed on Chart 2.

**Chart 2:** Reasons for having liked or not the PPE

Liked	Did not Like
Protects from loud noises when necessary	Does not hear the instrument played
Sound does not bother much	Prevents one from hearing the other musicians
The sound becomes smoother and more pleasant	Prevents from hearing the natural sound of the instrument
Better sensitivity	Much bothersome
Comfortable	Loss of hearing sensitivity
Protection and better sensitivity to singing	Makes it difficult to tune the instrument
Reduces sound pollution	Can not adapt
Ideal for intense music	Feeling of autophony of the sound played
Does no feel tinnitus at the end of exposure	Loses sound perception
	Inhibits the other sounds

There was no statistically significant difference related to the use or not of the hearing protection device for the following factors: age range, hearing loss, type.

## DISCUSSION

Of the 34 individuals who underwent part of this study, many of them perform or have performed in other musical scenarios, and also in other professional noisy activities or leisure noisy activities, adding to the hearing loss. The most commonly found hearing complaints in our study were: discomfort to sound 20 individuals (58.8%), tinnitus 16 (47.06%) and hearing loss 9 (25.71%), in agreement with the literature^7^,^21^, ^22^, ^23^.

When they were asked about the possibility of music causing hearing impairment, 27 individuals (77.14%) answered yes, although only 9 individuals (25.71%) stated they took some care regarding their hearings in performances with sound amplification, sound exposure in leisure activities and/or at work in factories. We observed that the individuals in the study did not know exactly how to protect their hearing, although they knew of the possibility of having a hearing impairment because of being exposed to loud music.

By analyzing the median values between the right and left ears we noticed a significant difference between the control and the experimental groups, suggesting the presence of hearing loss in the individuals who participated in the study who were exposed to music only. Such findings are in agreement with prior studies^6^,^7^,^20^, ^21^, ^22^.

Of the 23 individuals exposed to music only, 12 (52.1%) had hearing loss - more people than what was found in the previous work by this author involving the Municipal Band of Blumenal, with 13% of hearing loss^7^.

In other studies involving musicians, high rates of hearing loss were also observed. Among the members of the symphonic orchestra of Chicago, 42 people were found to have hearing impairment(71%)20. Of 21 musicians from varied rock bands, 11 (52,4%) had some hearing impairment^6^. Of 50 musicians from carnival frevo and maracatu music bands, 42.1% of the components of the Frevo band had hearing loss, and this rate was 16.1% for the members of the maracatu band21. In assessing the members of the Military Police Band of Santa Catarina, with special emphasis to the brass blowing instruments group - which were the most played instruments, 41% of them had hearing impairment22.

In the present investigation we measured the sound pressure levels during the collective rehearsal of the band, with mean values of 96.4 to 106.9 dB(A) SPL, with peaks of up to 110.9 dB(A) SPL, according to the aforementioned measurement arrangement.

In our literature review we noticed that a very complex procedure is necessary in order to measure sound pressure in music, because of its frequency and intensity variability and the level of sound the musicians are exposed to, which depends on exposure time, presentation site, collective and rehearsals, life style and other factors.

When factory workers are exposed to a sound pressure level above 85 dBH, it is known that they can develop hearing loss, depending on the length of exposure^23^. However, it is still unknown if the industry's standards are applicable to musicians, for the following reasons^1^,^2^,^6^:

§ in music, the predominant frequencies are low, less harmful because the stapes dampens the lower frequencies more effectively; in factories the noises are of higher frequencies;

§ in factories the noise is continuous throughout almost the entire day, while in music, the temporal pattern is floating, music is played for shorter periods, with certain periods of peak and pause between them, when the ear can rest and recover;

§ it is suggested that pleasurable sounds are less harmful than the undesired ones.

However, one has to take into account that the musician will be exposed to music during his or her entire professional career, and it is paramount that he or she has normal hearing. Any type of hearing loss is undesirable, because depending on its severity it may impair the individual's perception of some sounds and tones, affecting the sound balance between the instruments20,24,25.

In Brazil, we still do not have laws that protect musicians from the damage caused by loud music. The variability of opinions regarding the dangers of music to hearing makes it difficult to implement preventive actions related to music induced hearing loss (MIHL) to this type of professional.

Currently in Sweden, there are two recommendations for occupational safety limits associated with work and musical activities, both for musicians and listeners^18^.

In the Brazilian A.B.N.T (Brazilian Association of Technical Standards) standards there is nothing related to noise control in leisure activities^21^. It is also stressed that it is necessary to classify safety standards, and also the number of days of work allowed per week, the number of hours daily and the levels of sound pressure emitted during each performance.

The lack of legal standards for specific sound exposure for musicians may create this false impression that this type of work environment is free from auditory risks, because all musicians would benefit from a specific pattern for hearing protection^26^.

Of the 23 individuals exposed only to music in the group studied, 12 subjects (52.1%) had hearing loss, and also other auditory symptoms.

Numerous studies have shown the presence of music-induced hearing loss (MIHL), and also other hearing disorders among musicians with tinnitus, hypocusis or discomfort to loud sounds among others^7^,^17^,^19^,^22^,^25^,^27^, justifying the need for hearing protection programs in this industry.

In the literature we can find some strategies for preventing hearing loss induced by noise in this type of situation19:

§ Health appeal: Handouts about the harm caused by loud music to the human hearing, having the following as targets: schools and colleges, music and equipment stores, shopping malls, concert producers and promoters and medical centers.

§ Engineering controls: Keeping sound pressure level around 103dB(A) in concerts, by means of lining the walls with acoustic material, and avoiding powerful high frequency amplifiers.

§ Education: Educational programs for sound technicians about the risks associated with noise and measures to prevent hearing loss. Also, to encourage people to go to these places to have a 16 hour hearing rest after exposure to high levels of sound exposure.

§ Individual protection: concert organizers should provide disposable ear plugs in rock concerts. Rock concert fans should consider the use of customized hearing protection devices.

Hearing loss prevention among musicians still is a difficult goal to achieve, because musicians still see sound pressure levels recording in a very contradictory way, therefore they refuse to wear hearing protection^27^.

Laitinen (2005)^25^ performed a study with five orchestras, in order to find out how musicians dealt with this issue of hearing protection. The study showed that 94% of the participants were concerned with some hearing deficit, tinnitus, pain, stress reduction and fatigue. Notwithstanding, only 6% of those who participated in the study always wore their PPE. The author states that motivation and practice are necessary in order to increase the possibilities of musicians wearing hearing protection.

In plants and factories, campaigns are becoming increasingly more creative, using the language of the workers to talk about their day-to-day difficulties, using strategies such as calls, posters and plays^28^.

In the present investigation, the researcher was concerned with educating and training the band members regarding the use of hearing protection, as well as in bringing to them awareness about the risks of hearing impairment caused by periodic exposure to loud music, even then she encountered resistance regarding its use, as it has been recently reported by other authors^8^,^25^.

Many complaints presented in this study are similar to those reported by users of conventional hearing protection devices: it is difficult to understand others, difficult to hear the sound of their own instrument, it prevents communication, a feeling of isolation, effect of occlusion, among others. These were complaints reported by the band members who wore the hearing protection device with uniform sound damping used in the present investigation.

In the present study we may have had an effect of over-damping, that is, the protection may have provided damping above the levels needed by musicians, thus distorting the sound, bringing about this feeling of autophonia, caused by non-uniform damping to all the frequencies of the sound spectrum.

We did not observe levels of statistical significance for the following factors: hearing loss, age range of the subjects and type regarding the use or not of a hearing protection device.

## CONCLUSION

In the present investigation we noticed that the members of the municipal band of Indaial were aware of the risks associated with the exposure to loud music, however, they did not know how to protect themselves.

They seemed interested in trying the hearing protection device with uniform damping, however it was not effective because it was little used, both among those individuals with proven hearing loss detected during audiologic evaluation, as among those with hearing symptoms. We also noticed that many of the complaints reported by the subjects in this study were similar to those reported by users of conventional hearing protection devices, suggesting the possibility that the damping brought about by the protection used in the study have been higher that what was necessary, or even an effect of occlusion caused by the non-uniform damping among all frequencies of the sound spectrum. The individuals were split in regards to their continuing to wear the protection device in their professional careers. We believe that, by means of a systematic follow up of the group, periodic audiologic evaluation, new treatment and the use of other individually moldable hearing protection devices, the level of acceptability in the group could be altered. However, it is also necessary to have a law that encompasses all professionals associated with music, thus generating financial resources for the continuation of this study.
